# Supplementary material for: Association between fine particulate matter (PM2.5) and violence cases in South Korea: A nationwide time-stratified care-crossover study
Source: PLoS One. 2024 Dec 17;19(12):e0315914. doi: 10.1371/journal.pone.0315914 (PMC11651589; doi:10.1371/journal.pone.0315914)
Supplement: S1 File — (DOCX) [file pone.0315914.s001.docx]

**Supplementary Materials**

**Title: Association between fine particulate matter (PM_2.5_) and violence cases in South Korea: a nationwide time-stratified care-crossover study**

**Authors** Jiwoo Park*, Jieun Oh*, Hyewon Yoon, Ayoung Kim, Cinoo Kang, Dohoon Kwon, Jinah Park, Ho Kim, and Whanhee Lee

**Corresponding author** Whanhee Lee, School of Biomedical Convergence Engineering, Pusan National University, 49 Busandaehakro, Yangsan, South Korea, [whanhee.lee@pusan.ac.kr](mailto:whanhee.lee@pusan.ac.kr)

**1. Air Pollution Prediction Model**

This model was provided air pollution data by the AiMS-CREATE team (hereafter “the team”), which is a research network for environmental health between Pusan National University and Seoul National University in Korea, and their products were used in previously published studies.^1,2^

**(1) Information on monitoring station**

As response variables for the air pollution prediction modeling (i.e. as true values), we collected ground-level hourly measured PM_2.5_ concentrations from the Air Korea database provided by the Ministry of Environment (URL: <https://www.airkorea.or.kr/>) Korea from Jan. 01, 2015 to Dec 31, 2022. To reduce potential biases, we used concentration data from monitoring sites with observations for ≥ 9 months per year (observed 75% or over). The total number of selected monitoring sites was 483 for PM_2.5_ (105 stations in 2015 to 483 stations in 2022). From the selected monitoring sites, we calculated the average ambient concentrations of daily PM_2.5_.

**(2) Explanatory variables for prediction models with single machine learning algorithms**

**(2.1) Satellite-derived data**

Daily variables based on satellite remote sensing from 2015 through 2022 were used as major predictors. First, the team used a 1km^2^ grid cell shape file provided by Statistics Korea. Then, the team collected a total of 47 daily remote sensing variables through the Google Earth Engine (URL: <https://earthengine.google.com/>), and these variables included daily aerosol optical depth, meteorological, surface reflectance and landcover data from different satellite-based databases (the Table below). If the spatial resolution of certain variables was higher than 1km^2^, then we calculated and allocated the average values for each variable inside the boundary of each 1 km^2^ grid cell. Also, if the spatial resolution of certain variables was lower than 1 km^2^, we allocated the nearest values to the centroid of the 1 km^2^ grid cells and calculated the average values when two or more values were included in each 1 km^2^ grid cell. Then, we used the *missForest* method to impute missing values in a 1 km^2^ grid cell which were not measured with each satellite. Finally, we standardized the satellite-driven data to make zero-mean and unit-variance.

| **Data source** | **Predictor variables** | **Spatiotemporal**  **Resolution** |
| --- | --- | --- |
| ERA5-Land Daily Aggregated - ECMWF Climate Reanalysis | Temperature_2m | 11.13 km, Hourly |
|  | Skin_temperature |  |
|  | Soil_temperature_level_1 |  |
|  | Leaf_area_index_low_vegetation |  |
|  | Leaf_area_index_high_vegetation |  |
|  | Total_precipitation |  |
|  | Surface_pressure |  |
|  | u_component_of_wind_10m |  |
|  | v_component_of_wind_10m |  |
| MOD09GA.061 Terra Surface Reflectance Daily Global 1km and 500m | Sur_refl_b01 | 500 m, Daily |
|  | Sur_refl_b02 |  |
|  | Sur_refl_b03 |  |
|  | Sur_refl_b07 |  |
| MOD11A1.061 Terra Land Surface Temperature and Emissivity Daily Global 1km | Emis_31 | 1 km, Daily |
|  | Emis_32 |  |
|  | LST_Night_1km |  |
|  | LST_Day_1km |  |
| CFSV2: NCEP Climate Forecast System Version 2, 6-Hourly Products | Maximum_specific_humidity_at_2m_height_above_ground_6_hour_interval | 22.26 km, 6-hour |
|  | Minimum_specific_humidity_at_2m_height_above_ground_6_hour_interval |  |
|  | Specific_humidity_height_above_ground |  |
|  | Maximum_temperature_height_above_ground_6_hour_interval |  |
|  | Minimum_temperature_height_above_ground_6_hour_interval |  |
|  | Geopotential_height_surface |  |
| MCD19A2.061: Terra & Aqua MAIAC Land Aerosol Optical Depth Daily 1km | Optical_Depth_047 | 1 km, Daily |
|  | Optical_Depth_055 |  |

**Meteorological variables:** Air pollution can be affected spatially and temporally by meteorological factors such as temperature, wind speed and direction, precipitation, humidity, and cloud droplets, the team collected satellite datasets from the 5^th^ generation European Center for Medium-Range Weather Forecasts atmospheric reanalysis (ERA5)^3^, which is an archive at the Google Earth Engine. The ERA5 data (~9 to 11km^2^) includes average air temperature at 2m height (daily average), skin temperature (daily average), soil temperature in layer 1 (daily average), total precipitation (daily sums), surface pressure (daily average), 10m u-component of wind (daily average), and 10m v-component of wind (daily average).

Additionally, from the National Centers for Environmental Prediction (NCEP) Climate Forecast System (CFS), we collected maximum/minimum/mean specific humidity at 2m height (6-hour average), maximum/minimum temperature at 2m height (6-hour average) and geopotential height at the surface (6-hour average). Also, from the Terra Moderate Resolution Imaging Spectroradiometer (MODIS), we used daytime/nighttime land surface temperature (daily average), emissivity with bands 31 and 32 (daily average), and surface reflectance with bands 1,2,3 and 7 (daily average).

**Aerosol variables:** The team used two aerosol optical depth variables: Aerosol optical depth over land retrieved in the MODIS Green band (0.55 μm) and aerosol optical depth over land retrieved in the MODIS blue band (0.47 μm). Aerosol optical depth is not received at high altitudes (greater than 4.2 km) except when smoke or dust is detected; rather, this value reports a static value of 0.02 used for atmospheric correction.

**Vegetation variables:** The variables related to the vegetation in each grid were collected in the following two datasets in Google Earth Engine. With the ERA5 dataset, we collected one-half of the total green leaf area per unit horizontal ground surface area for high vegetation type (daily average) and one-half of the total green leaf area per unit horizontal ground surface area for low vegetation type (daily average). Also, with the MODIS dataset, we used the 16-day averaged Enhanced Vegetation Index (EVI), the second vegetation layer that can mitigate canopy background variations, preserve sensitivity in dense vegetation, and utilize the blue band to eliminate residual atmosphere contamination from smoke and sub-pixel thin cloud clouds.

**(2.2) Regional data**

**Population density:** We used population density data from the community health-related factor database sourced from the Korea Centers for Disease Control and Prevention, which comprises data pertaining to health determinants encompassing physical, environmental, and demographic factors influencing community health levels and disparities. Therefore, as population density data was collected at a Korean district level (‘Si-gun-gu’) and its spatial resolution is bigger than 1km^2^, the same value was assigned to the grids that were included in each district.

**(3) Machine learning modeling and model ensemble**

Three machine learning-based models, namely random forest, light gradient boosting, and deep neural network, were used to predict daily PM_2.5_ averages (individually) with a 1 km^2^ grid during 2015–2022. The team trained the algorithm individually on all input variables (daily satellite variables with EVI and population density variables) and parameters of each algorithm selected by cross-validated grid search. To avoid overfitting, we validated each model with a cross-validation. In addition, because the number of monitoring stations in Korea has increased every year during the study period, we did a year-stratified cross-validation: training with 80% of the data and testing a prediction performance at the remaining 20% of data by each study year (from 2015 to 2022). From these procedures, we found the optimized model for each algorithm and calculated 1km^2^ daily predicted PM_2.5_ concentrations.

Furthermore, to increase the prediction performance, we performed an ensemble approach using the generalized additive model (GAM) to incorporate these three algorithms.^3^ This GAM model allows us to address a flexible regression for the monitoring concentrations against the estimates from each machine learning algorithm, and annual land-use variables by thin-plate splines. Here, we considered annual land-use variables collected from MODIS through the Google Earth Engine (the Table below)

| **Data source** | **Predictor variables** | **Collection Period** | **Spatiotemporal**  **Resolution** |
| --- | --- | --- | --- |
| Copernicus Global Land Cover Layers: CGLS-LC100 Collection 3 | Bare-coverfraction | 2015~2019 | 100m |
|  | Crops-coverfraction |  |  |
|  | Grass-coverfraction |  |  |
|  | Shrub-coverfraction |  |  |
|  | Tree-coverfraction |  |  |
|  | Urban-coverfraction |  |  |
|  | Water-permanent-coverfraction |  |  |
|  | Water-seasonal-coverfraction |  |  |
|  | Forest_type |  |  |
| GlobCover: Global Land Cover Map | Landcover | 2009~2010 | 300m |
| MCD12Q1.061 MODIS Land Cover Type Yearly Global 500m | LC_Type1 | 2002~2021 | 500m, Yearly |
|  | LC_Type2 |  |  |
|  | LC_Type3 |  |  |
|  | LC_Type4 |  |  |
|  | LC_Type5 |  |  |
|  | LC_Prop1 |  |  |
|  | LC_Prop2 |  |  |
|  | LC_Prop3 |  |  |
|  | LC_Prop1_Assessment |  |  |
|  | LC_Prop2_Assessment |  |  |
|  | LC_Prop3_Assessment |  |  |
| MOD13A2.061 Terra Vegetation Indices 16-Day Global 1km | EVI | 2002~2020 | 1km, 16-day |

**Land-use variables:** To consider regional heterogeneity, we collected several land-use variables. From the Copernicus Global Land Service (CGLS), we used forest type with tree percentage vegetation cover bigger than 1% and percent vegetation cover for the various land cover classes including bare-sparse-vegetation, cropland, herbaceous vegetation, shrubland, forest, build-up, permanent water, and seasonal water. Also, with the global land cover map based on ENVISAT's Medium Resolution Imaging Spectrometer (MERIS) Level 1B data, we applied the land cover map data.

In addition, there are the yearly land-use data provided by the MODIS dataset. The data contains the annual International Geosphere-Biosphere Programme (IGBP) classification, annual University of Maryland (UMD) classification, annual Leaf Area Index (LAI) classification, annual BIOME-Biogeochemical Cycles (BGC) classification, annual Plant Functional Types classification, LCCS1 land cover layer (confidence), LCCS1 land cover layer (confidence) and LCCS3 surface hydrology layer (confidence). As the data provides yearly data, we assigned the same value to the daily values that were included in each year.

**(4) Performance of the GAM Ensemble Prediction Model**

Among the three pollutant models with a single algorithm and the GAM ensemble model, the GAM ensemble model showed the best prediction accuracy for PM_2.5_. Thus, we summarized the performance of the GAM ensemble model below: **Table S1-S3**

**2. Supplementary Tables**

**Table S1. Summary statistics on the performance of the PM_2.5_ ensemble prediction model during the study period (2015 to 2019).** RMSE: root mean squared error, MAE: mean absolute error. RMSE and MAE are in the unit of PM_2.5_ (μg/m^3^).

|  |  | **R^2^** | **RMSE** | **MAE** | **Mean** |
| --- | --- | --- | --- | --- | --- |
| **PM_2.5_** | **Total years** | 0.944 | 3.219 | 2.187 | 23.71 |
|  | **2015** | 0.889 | 3.375 | 2.179 | 25.74 |
|  | **2016** | 0.882 | 3.352 | 2.290 | 25.41 |
|  | **2017** | 0.925 | 3.283 | 2.258 | 23.74 |
|  | **2018** | 0.957 | 3.453 | 2.148 | 21.71 |
|  | **2019** | 0.965 | 3.135 | 2.138 | 21.97 |

**Table S2. Season-specific test R2 compared to the concentration values from the monitoring stations.** Spring: March to May, Summer: June to August, Autumn: September to November, and Winter: December to February

| **Years** | **Spring** | **Summer** | **Autumn** | **Winter** |
| --- | --- | --- | --- | --- |
| **R^2^** | 0.958 | 0.934 | 0.949 | 0.965 |

**Table S3. Monthly test R2 compared to the concentration values from the monitoring stations**

| **Month** | **1** | **2** | **3** | **4** | **5** | **6** | **7** | **8** | **9** | **10** | **11** | **12** |
| --- | --- | --- | --- | --- | --- | --- | --- | --- | --- | --- | --- | --- |
| **R^2^** | 0.967 | 0.963 | 0.968 | 0.933 | 0.941 | 0.930 | 0.931 | 0.924 | 0.925 | 0.930 | 0.952 | 0.963 |

**Table S4. Descriptive information on violence cases among Korea National Hospital Discharge In-depth Injury Survey Data during the study period (2015–2019) in South Korea by year.**

|  |  | **Year** | | | | | |
| --- | --- | --- | --- | --- | --- | --- | --- |
|  |  | **Total** | **2015** | **2016** | **2017** | **2018** | **2019** |
| **Total** |  | 561 (100.0%) | 544 (100.0%) | 612 (100.0%) | 578 (100.0%) | 572 (100.0%) | 2867 (100.0%) |
| **Sex** | **Males** | 373 (66.49%) | 358 (65.81%) | 410 (66.99%) | 417 (72.15%) | 379 (66.26%) | 1937 (67.56%) |
|  | **Females** | 188 (33.51%) | 186 (34.19%) | 202 (33.01%) | 161 (27.85%) | 193 (33.74%) | 930 (32.44%) |
| **Age** | **0-19 years** | 530 (94.47%) | 511 (93.93%) | 569 (92.97%) | 537 (92.91%) | 513 (89.69%) | 2660 (92.78%) |
|  | **20-39 years** | 70 (12.48%) | 90 (16.54%) | 100 (16.34%) | 97 (16.78%) | 85 (14.86%) | 442 (15.42%) |
|  | **40-64 years** | 186 (33.16%) | 200 (36.76%) | 228 (37.25%) | 206 (35.64%) | 203 (35.49%) | 1023 (35.68%) |
|  | **65-79 years** | 274 (48.84%) | 221 (40.62%) | 241 (39.38%) | 234 (40.48%) | 225 (39.34%) | 1195 (41.68%) |
|  | **80+ years** | 31 (5.53%) | 33 (6.07%) | 43 (7.03%) | 41 (7.09%) | 59 (10.31%) | 207 (7.22%) |
|  | **0-64 years** | 27 (4.81%) | 30 (5.51%) | 35 (5.72%) | 34 (5.88%) | 48 (8.39%) | 174 (6.07%) |
|  | **65+ years** | 4 (0.71%) | 3 (0.55%) | 8 (1.31%) | 7 (1.21%) | 11 (1.92%) | 33 (1.15%) |
| **Holiday** | **Yes** | 26 (4.63%) | 16 (2.94%) | 23 (3.76%) | 20 (3.46%) | 21 (3.67%) | 106 (3.7%) |
|  | **No** | 535 (95.37%) | 528 (97.06%) | 589 (96.24%) | 558 (96.54%) | 551 (96.33%) | 2761 (96.3%) |
| **Urban** | **Yes** | 498 (88.77%) | 490 (90.07%) | 548 (89.54%) | 527 (91.18%) | 505 (88.29%) | 2568 (89.57%) |
|  | **No** | 63 (11.23%) | 54 (9.93%) | 64 (10.46%) | 51 (8.82%) | 67 (11.71%) | 299 (10.43%) |
| **GRDP per capita** | **High** | 255 (45.45%) | 261 (47.98%) | 291 (47.55%) | 268 (46.37%) | 236 (41.26%) | 1311 (45.73%) |
|  | **Low** | 306 (54.55%) | 283 (52.02%) | 321 (52.45%) | 310 (53.63%) | 336 (58.74%) | 1556 (54.27%) |

**Table S5. Descriptive information on violence cases among Korea National Hospital Discharge In-depth Injury Survey Data during the study period (2015–2019) in South Korea by season and mechanism of violence case.** ICD-9: International Statistical Classification of Diseases and Related Health Problems 9th Revision.

| **Mechanism of violence** | **ICD-9 codes** | **Total seasons** | **Warm seasons** | **Cold seasons** |
| --- | --- | --- | --- | --- |
| Total | X85-Y09 | 2867 (100%) | 1549 (100%) | 1318 (100%) |
| Collision | Y00, X96, Y04-Y05, Y07 | 2543 (88.7%) | 1359 (87.7%) | 1184 (89.8%) |
| Laceration, Puncture, Cut | X99 | 173 (6%) | 99 (6.4%) | 74 (5.6%) |
| Others | X85-X98, Y01-03, Y06, Y08 | 121 (4.2%) | 73 (4.7%) | 48 (3.6%) |
| Unknown | - | 30 (1%) | 18 (1.2%) | 12 (0.9%) |

**Table S6. Descriptive information on violence cases among Korea National Hospital Discharge In-depth Injury Survey Data during the study period (2015–2019) in South Korea by season and treatment result.**

| **Treatment result** | **Total seasons** | **Warm seasons** | **Cold seasons** |
| --- | --- | --- | --- |
| **Total** | 2867 (100%) | 1549 (100%) | 1318 (100%) |
| **Recovered (Complete recovery and Partial recovery)** | 2757 (96.16%) | 1492 (96.32%) | 1265 (95.98%) |
| **Diagnosed but not treated** | 48 (1.67%) | 24 (1.55%) | 24 (1.82%) |
| **Not recovered (No improvement)** | 45 (1.57%) | 26 (1.68%) | 19 (1.44%) |
| **Discharged due to death** | 16 (0.56%) | 7 (0.45%) | 9 (0.68%) |
| **Others** | 1 (0.03%) | 0 (0.00%) | 1 (0.08%) |

**Table S7. Associations between short-term exposure to PM_2.5_ (lag 0–2) and violence cases by season and age group.**

| **Seasons** | **Age group** | **OR (95% CI)** |
| --- | --- | --- |
| **Every season** | Total | 1.07 (1.02, 1.12) |
|  | 0-19 years | 1.09 (0.96, 1.24) |
|  | 20-39 years | 1.11 (1.03, 1.20) |
|  | 40-64 years | 1.02 (0.95, 1.11) |
|  | 65-79 years | 1.11 (0.89, 1.39) |
|  | 80+ years | 1.75 (0.96, 3.19) |
| **Warm seasons** | Total | 1.05 (0.96, 1.15) |
|  | 0-19 years | 1.04 (0.83, 1.30) |
|  | 20-39 years | 1.18 (1.01, 1.37) |
|  | 40-64 years | 0.99 (0.86, 1.14) |
|  | 65-79 years | 0.87 (0.59, 1.28) |
|  | 80+ years | 0.84 (0.29, 2.46) |
| **Cold seasons** | Total | 1.08 (1.02, 1.14) |
|  | 0-19 years | 1.09 (0.94, 1.28) |
|  | 20-39 years | 1.09 (0.99, 1.20) |
|  | 40-64 years | 1.03 (0.94, 1.13) |
|  | 65-79 years | 1.30 (0.98, 1.72) |
|  | 80+ years | 2.52 (1.04, 6.12) |

**Table S8. Sensitivity analysis.**

|  |  | **OR (10 µg/m^3^ increase)** | **LCL** | **UCL** | **P** |
| --- | --- | --- | --- | --- | --- |
| **PM_2.5_** | **Lag 0** | 1.04 | 1 | 1.08 | 0.064 |
|  | **Lag 1** | 1.05 | 1.01 | 1.09 | 0.018 |
|  | **Lag 2** | 1.05 | 1.01 | 1.09 | 0.014 |
|  | **Lag 3** | 1.03 | 0.99 | 1.07 | 0.203 |
|  | **Moving Average 0-1** | 1.06 | 1.01 | 1.11 | 0.019 |
|  | **Moving Average 0-2** | 1.07 | 1.02 | 1.12 | 0.007 |
|  | **Moving Average 0-3** | 1.07 | 1.02 | 1.13 | 0.009 |
| **Temperature** | **Lag 0-3** | 1.07 | 1.02 | 1.13 | 0.005 |
|  | **Lag 0-7** | 1.08 | 1.02 | 1.13 | 0.004 |
|  | **df=3 for exposure-response relationship** | 1.07 | 1.02 | 1.12 | 0.006 |
|  | **df=5 for exposure-response relationship** | 1.07 | 1.02 | 1.12 | 0.006 |

**References for the Supplementary Materials**

1. Kim Y, Oh J, Kim S, et al. Relationship between short-term ozone exposure, cause-specific mortality, and high-risk populations: a nationwide, time-stratified, case-crossover study. *Environmental Research* 2024: 119712.

2. Min J, Lee W, Kang D-H, et al. Air pollution and acute kidney injury with comorbid disease: A nationwide case-crossover study in South Korea. *Environmental Research* 2024; **260**: 119608.

3. Di Q, Amini H, Shi L, et al. An ensemble-based model of PM2.5 concentration across the contiguous United States with high spatiotemporal resolution. *Environment International* 2019; **130**: 104909.
