# Supplementary material for: Association between fine particulate matter (PM2.5) and violence cases in South Korea: A nationwide time-stratified care-crossover study
Source: PLoS One. 2024 Dec 17;19(12):e0315914. doi: 10.1371/journal.pone.0315914 (PMC11651589; doi:10.1371/journal.pone.0315914)
Supplement: Attachment — Submitted filename: Responses to Reviewers_20241025.docx [file pone.0315914.s002.docx]

**Dear Editor,**

We sincerely thank the reviewers for their insightful comments on our manuscript. We have carefully revised the manuscript and responded to each comment point by point below.

**Reviewer #1: Park et al. evaluated the association of PM_2.5_ with violence using a time-stratified case-crossover design and a conditional logistic regression. The manuscript is confusing and needs some modifications as follows:**

**#1.1. In many sections, the authors said that they predicted PM_2.5_, and it is not clear whether they did some imputations or just used the predicted values.**

**[Response]** Thank you for your comment and our apologies for the confusion. In this study, we used “*predicted* values of PM_2.5_ concentrations”, which was driven by a machine-learning ensemble prediction model to cover unmonitored areas. In other words, this model predicted daily PM_2.5_ concentrations for both monitored and unmonitored areas during the study period, recent studies have adopted the modeled air pollution concentrations to provide nationwide risk estimates.^1-3^ To clarify it, we have revised the related text (lines 123-133 on page 7) and have included detailed explanations and performance for the PM_2.5_ prediction model in the Supplemental Materials “1. Air pollution prediction models” and Table S1.

**(Lines 123-130 on Page 7)**

To cover unmonitored districts, a nationwide daily modeled PM_2.5_ (the predicted value using a machine-learning ensemble prediction model with a 1 km^2^ spatial resolution) was used as an exposure for all districts. The modeled PM_2.5_ (24-hour average) was provided by the AiMS-CREATE team, and their prediction models were used in previous studies.^18,19^ The ensemble model incorporates three machine-learning algorithms (random forest, extreme gradient boosting, and deep neural network). Detailed information on the model is reported in the Supplementary Materials, “1. Air pollution prediction models” and Supplementary Table 1 (Table S1).

**#1.2. There are several over-interpretations. This study found some associations between short-term PM_2.5_ exposure and the risk of violence. Some sentences, such as "6.21% of the excess violence was due to non-compliance with the WHO guidelines (daily PM_2.5_ > 15 µg/m^3^)" and "Our generalizable findings", seems to be over-interpreted.**

**[Response]** We agree with your comments and acknowledge that there is a possibility of misinterpretation. Thus, we have revised related sentences across the manuscript (main change: we used “related or associated” instead of “attributable” to avoid misinterpretation). We would be glad if you could consider our modifications below:

**(Lines 267-268 on Page 14)**

Approximately 6.21% (1.59–10.33) and 1.07% (0.28–1.78) of excess violence cases were related to non-compliance with the WHO air quality guidelines (15 µg/m^3^) and NAAQS (35 µg/m^3^), respectively.

**(Lines 288-289 on Page 20)**

We also found that the excess fractions of violence associated with non-compliance with WHO guidelines and NAAQS and KAS were 6.21% and 1.07%, respectively.

**(Lines 34-37 on Page 2; Abstract)**

The estimated excess fraction of violence cases related to PM_2.5_ was 14.53% (95% CI: 4.54%–22.92%), and 6.42% (95% CI: 1.97%–10.26%) of the excess violence was related to non-compliance with the WHO guidelines (daily PM_2.5_ > 15 µg/m^3^). Our findings might be the evidence for the need of establishing elaborate action plans and stricter air quality guidelines to reduce the hazardous impacts of PM_2.5_ on violence in South Korea.

**#1.3. There are too less information in the Figure and Table legends. At least, what variables were used for the adjustments, what methods were used, and the acronyms need to be explained. In addition, what results are adjusted for daily mean temperature?**

**[Response]** Our apologies for the lack of information on figures and tables. We have added detailed information to all figures and tables as below:

**Figure 2. Flexible association between short-term exposure to PM_2.5_ and violence cases.** Each Blue and Green dashed lines indicate WHO: World Health Organization air quality guidelines (daily average PM_2.5_: 15 µg/m^3^), NAAQS: National Ambient Air Quality Standards (daily average PM_2.5_: 35 µg/m^3^) in the United States, and KAS: Korean Air Quality Standards (daily average PM_2.5_: 35 µg/m^3^), respectively. A conditional logistic regression model, adjusted for holiday and daily mean temperatures, within a time-stratified case-crossover design was used to estimate odds ratios and 95% confidence intervals. We classified the study districts (si/gun/gu) into urban (si and gu) and rural (gun) districts. GRDP High and Low areas are separated by a median value.

**Figure 3. Associations between short-term exposure to PM_2.5_ (lag 0–2) and violence cases by season and subgroup.** OR: Odds ratio per 10 µg/m^3^ of PM_2.5_. A conditional logistic regression model, adjusted for holiday and daily mean temperatures, within a time-stratified case-crossover design was used to estimate odds ratios and 95% confidence intervals. Wald type test was used to test the effect modification. We classified the study districts (si/gun/gu) into urban (si and gu) and rural (gun) districts. GRDP High and Low areas are separated by a median value.

**Table 2. Excess numbers and fractions of violence cases attributable to PM_2.5_ and non-compliance with the current WHO air quality guidelines and NAAQS in the United States**. WHO: World Health Organization, NAAQS: National Ambient Air Quality Standards. KAS: Korean Air Quality Standards. We classified the study districts (si/gun/gu) into urban (si and gu) and rural (gun) districts. GRDP High and Low areas are separated by a median value.

In addition, yes, we adjusted daily mean temperatures as a confounder. The daily average temperatures are generally considered as a confounder, which could affect both the daily mean PM_2.5_ (our main exposure) and the daily violence counts (outcome),^4,5^ when examining the association between air pollutants and health outcomes. Therefore, for consistency, we also controlled for daily mean temperatures in this study as previous studies performed.^6,7^

**#1.4. Line 225, In particular, Particularly,**

**[Response]** Thank you for pointing it out. We have revised the text.

**#1.5. "Estimation of the excess violence burden attributable to PM_2.5_" is highly confusing. "We calculated daily attributable estimates, and the sum of daily attributable violence cases represents the total excess violence cases". The authors have calculated daily average of violence cases, and when the number of cases is above the average, then it is considered as the excess violence cases. What is the purpose of these results? I think it only causes confusing.**

**[Response]** We appreciate your careful comments. First, we used the daily sum of violence cases (counts) and calculated excess violence counts related to daily PM_2.5_ using the corresponding odd ratio (OR) for the daily PM_2.5_.^8,9^ Through this procedure, we ultimately aimed to quantify and present “*the excess burden*” of violence related to “the whole range of PM_2.5_ during the entire study period” (i.e., the actual/quantitative impact of the exposure) because OR could provide only the risk ratio per unit increase of PM_2.5_. This information is critical for the planning and evaluation of public health interventions, and it is better provided by relative excess measures such as the attributable fraction (AF), or absolute excess measures such as the attributable number (AN).^10^ To clarify it, we have revised the related texts with the necessity of the excess measures and detailed calculation procedures. Please see below:

**(Lines 194-210 on Pages 10-11)**

**Excess violence burden related to PM_2.5_**

Odd ratio estimates for short-term exposure to PM_2.5_ were translated into excess violence cases related to PM_2.5_, to demonstrate the excess burden due to PM_2.5_ exposures during the study period. The estimates of the excess burden (either as an attributable burden or relative excess measures of outcomes) are widely used to assess the actual and quantitative public health impacts of extreme exposures (e.g., heatwaves or high-level air pollution).

To calculate it, we created district-specific time-series datasets including daily mean PM_2.5_ and daily counts of violence. For each time-series data, we calculated the daily excess violence cases attributable to PM_2.5_ using the corresponding estimated ORs for the PM_2.5_ concentrations of each day (daily excess violence cases related to daily PM_2.5_: ${sum of violecne cases}_{t} *[{OR}_{t}-1]/{OR}_{t}$; where *t* indicates the day, and OR_t_ presents the daily OR for the PM_2.5_ concentrations of day *t*). The sum of daily excess violence cases indicates the total excess violence cases related to PM_2.5_ during the study period, and its ratio with the total number of violence provides the total excess fraction (%) of violence cases related to short-term PM_2.5_ exposures.

**#1.6. No interaction tests were done and there are some sentences about more prominent association of PM_2.5_ with violence incidence in males and younger populations.**

**[Response]** We sincerely appreciate your comment. In accordance with your suggestion, we have tested the effect modification of the association between PM_2.5_ and violence cases using the Wald test, and the results have been added to the revised Figure 3. Further, we described the statistically meaningful results in the main text.

**(Lines 255-262 on Pages 13-14)**

Figure 3 shows the association between PM_2.5_ and violence in the total population and by sub-group. In the total population, the association between PM_2.5_ and violence was evident with an OR of 1.07 with 95% CI: 1.02–1.12. The association was weakly higher in rural areas (OR: 1.23, 95% CI: 1.02–1.48) than in urban areas (OR: 1.06, 95% CI: 0.99–1.12) (Wald test p-value: 0.096). Further, the association between PM_2.5_ and violence was statistically pronounced in cold seasons (OR: 1.08, 95% CI: 1.02–1.14) compared to warm seasons (1.05, 0.96–1.15), and the association was more pronounced in people aged 65 (OR:1.33, 95% CI: 1.04–1.71) or older than those aged 64 or less in cold seasons (Wald test p-value: 0.042).

**(Revised) Figure 3. Associations between short-term exposure to PM_2.5_ (lag 0–2) and violence cases by season and subgroup.** OR: Odds ratio per 10 µg/m^3^ of PM_2.5_. A conditional logistic regression model, adjusted for holiday and daily mean temperatures, within a time-stratified case-crossover design was used to estimate odds ratios and 95% confidence intervals. Wald type test was used to test the effect modification. We classified the study districts (si/gun/gu) into urban (si and gu) and rural (gun) districts. GRDP High and Low areas are separated by a median value.

**Reviewer #2: Thank you for the opportunity to review this manuscript. This study aims to evaluate the association between fine particulate matter (PM2.5) and violence incidence in South Korea using a case-crossover design. While I find the study's findings significant, there are several points where additional explanations and analyses could strengthen the results.**

**My major concerns are as follows:
There is considerable overlap and redundancy between the information provided in the supplementary files and the main text. Particularly, details related to the Data Source, Outcome Definition, Study Design, Statistical Analysis, and Estimation of the Excess Violence are spread across both sections. I recommend including these key elements more clearly in the main text.**
**[Response]** Thank you for your careful comments. Following your suggestion, we have tried to re-organize our manuscripts to avoid overlap and redundancy. Please see our revised files. We truly appreciate your opinion.

**#2.1. Lines 130-137: The authors should explain why they chose lag0-2 for their analysis.**

**[Response]** We are thankful for your feedback. Since violence cases usually occur as acute events, we adopted two days as a lag period based on previous relevant studies ^11-13^. We also thought the selection could increase the comparability with existing findings. We added more rationales and explanations for the lag period in the methods section like below:

**(Lines 164-166 on Page 9)**

For the main model, we selected a mean value of lag 0 to lag 2 PM_2.5_ exposure to address the average health risks associated with the same and the previous days’ exposures based on existing relevant studies.^21,31,32^

**(Lines 171-173 on Page 9)**

The relationship between relatively short-term temperature (one or two lag days) and violence, suicide, or other acute mental disorders has been identified in many related epidemiological studies.^13-16^

Additionally, we have performed sensitivity analyses regarding different lag days to check the robustness of our results, and the results have been added to Table S8. Our main results were consistent with different lag periods.

**#2.2. Lines 162-164: The explanation provided here is insufficient. Could the authors expand on this section?**

**[Response]** We greatly appreciate your comment. We have added more detailed explanations for the sensitivity analyses in the methods section below:

**(Lines 226-232 on Page 12)**

We performed a series of sensitivity analyses to examine whether our results were consistent with additional different modeling specifications in the total population. Sensitivity analyses were carried out about two aspects. First, we tried to show the robustness of PM_2.5_’s lag period. Single lag from 0 to 3 was executed and the moving average from lag 0-1 to 0-3 was tested. Second, for daily temperature, which was used as a covariate, we applied 3 and 7 days of lag period, and 3 and 5 degrees of freedom for an exposure-response relationship.

**#2.3. Lines 139-142: Could the authors consider further stratifying the age groups, similar to what was presented in Table 1 (e.g., 0-19, 20-39, 40-64, 65-79, 80+)? I believe there may be differences across age groups and occupations.**

**[Response]** We would like to express our gratitude for your meaningful comment. As you pointed out, we have added more specific results for stratifying age groups in Table S7. We found that the findings were consistent with the main results; i.e., a statistically significant association was found in the younger age group (20–39 years) rather than the older age groups (65–79 years and 80+ years) in total seasons, and the risk in the older age groups (65–79 years and 80+ years) was greater in the cold seasons than in the warm seasons. Therefore, we have added a sentence in the result section as follows:

**(Lines 262-263 on Page 14)**

This pattern remained consistent even when age groups were further subdivided (Table S7).

**Table S7. Associations between short-term exposure to PM_2.5_ (lag 0–2) and violence cases by season and age group.**

| **Seasons** | **Age group** | **OR (95% CI)** |
| --- | --- | --- |
| **Every season** | Total | 1.07 (1.02, 1.12) |
|  | 0-19 years | 1.09 (0.96, 1.24) |
|  | 20-39 years | 1.11 (1.03, 1.20) |
|  | 40-64 years | 1.02 (0.95, 1.11) |
|  | 65-79 years | 1.11 (0.89, 1.39) |
|  | 80+ years | 1.75 (0.96, 3.19) |
| **Warm seasons** | Total | 1.05 (0.96, 1.15) |
|  | 0-19 years | 1.04 (0.83, 1.30) |
|  | 20-39 years | 1.18 (1.01, 1.37) |
|  | 40-64 years | 0.99 (0.86, 1.14) |
|  | 65-79 years | 0.87 (0.59, 1.28) |
|  | 80+ years | 0.84 (0.29, 2.46) |
| **Cold seasons** | Total | 1.08 (1.02, 1.14) |
|  | 0-19 years | 1.09 (0.94, 1.28) |
|  | 20-39 years | 1.09 (0.99, 1.20) |
|  | 40-64 years | 1.03 (0.94, 1.13) |
|  | 65-79 years | 1.30 (0.98, 1.72) |
|  | 80+ years | 2.52 (1.04, 6.12) |

However, due to the limited data availability (the surveillance system database we used in this study does not include the information on enrollees’ occupation and income status), we were unable to consider an individual's occupation, and we have addressed this issue as a major limitation in the discussion section:

**(Lines 378-381 on Page 24)**

Third, due to the limited data availability, we were unable to explore the associations between PM_2.5_ and violence by individual socioeconomic factors, such as occupation or income levels, although these socioeconomic factors could be substantially associated with violence.

**#2.4. Table 1: I recommend presenting the cases by year and region.**

**[Response]** We are grateful for your comment. We have included the descriptive information about regions using the urban/rural indicator and gross regional domestic product (GRDP) per person in the revised Table 1. Additionally, we have examined the descriptive information by year and found no significant differences in the distributions and added the results to Table S4.

**Table 1. Descriptive information on violence cases among Korea National Hospital Discharge In-depth Injury Survey Data during the study period (2015–2019) in South Korea.** Holidays include Official public holidays in Korea. We classified the study districts (si/gun/gu) into urban (si and gu) and rural (gun) districts. GRDP High and Low areas are separated by a median value.

|  |  | **N** | **%** |
| --- | --- | --- | --- |
| **Total** |  | 2867 | 100 |
| **Sex** | **Males** | 1937 | 67.6 |
|  | **Females** | 930 | 32.4 |
| **Age** | **0-64 years** | 2660 | 92.8 |
|  | 0-19 years | 442 | 15.4 |
|  | 20-39 years | 1023 | 35.7 |
|  | 40-64 years | 1195 | 41.7 |
|  | **65+ years** | 207 | 7.2 |
|  | 65-79 years | 174 | 6.1 |
|  | 80+ years | 33 | 1.2 |
| **Holiday** | **Yes** | 106 | 3.7 |
|  | **No** | 2761 | 96.3 |
| **Urban/Rural** | **Urban** | 2568 | 89.6 |
|  | **Rural** | 299 | 10.4 |
| **GRDP per person** | **High** | 1311 | 45.7 |
|  | **Low** | 1556 | 54.3 |

**#2.5. How did the authors account for regional variations in their statistical analyses? I believe there could be differences based on geographical regions. If possible, I suggest conducting stratified analyses based on individual or regional income levels, followed by meta-analyses by city as part of a sensitivity analysis.**

**[Response]** Thank you for your comment. First, as you mentioned, addressing potential regional-level confounders/covariates is an important point in epidemiology studies. Nevertheless, we would like to carefully remind you that this study adopted the time-stratified case-crossover design to control the unmeasured confounders/covariates that are time-invariant or not markedly changed within a month.^11^ In other words, we set a case day as the date of violence for individual violence case and matched control days as days with the same day of the week within the same month in the same year. This time-stratified self-matching could control confounders that are time-invariant or do not change remarkably in a month, such as age, sex, weight, diet, and district-level regional variables like population density, gross regional domestic product, and other socio-environmental factors including park and medical accessibilities, population composition, and access to grocery shops.^11^ Due to these benefits, the time-stratified case-crossover design has been widely used in studies evaluating the risk estimates of short-term environmental exposure to health.^11,14-17^ Therefore, we would like to inform you that our risk estimates based on the time-stratified case-crossover design are the marginal risk estimates after considering the confounders, and they were not affected by regional-level characteristics unless the characteristics changed substantially within a month.

Nevertheless, we agree with your comment that stratified analyses by regional indicators could provide informative results to find vulnerable populations. Therefore, we collected two representative regional indicators that might cover district-level economic status, socioeconomic or medical infrastructures, and accessibility to medical services comprehensively: urban/rural indicator and GRDP (Gross Regional Domestic Product) per person for each district. Previous related studies reported that especially in South Korea, the urbanicity level is closely related to socioeconomic/medical characteristics.^18,19^ We collected annual district-level GRDP per 100,000 persons from 2015 to 2019 from Statistics Korea. Then, we calculated the district-specific average GRDP per 100,000 persons during the study period.

First, regarding urban/rural classification, we classified all study districts (si/gun/gu) into urban and rural districts, based on the Local Autonomy Act of Korea, which has been used in previous studies ^20,21^. The ‘si’ and ‘gu’ districts are defined as urban areas (in general, districts with a population of more than 50,000), and the ‘gun’ districts, which do not satisfy the criteria of ‘si’ or ‘gu’, were classified as rural areas. Second, we divided study districts into two categories (high/low) based on the median value of average GRDP per 100,000 persons. Then, we evaluated the association between PM_2.5_ and violence for each category via stratified analyses.

We found that rural areas and areas with low GRDP per 100,000 people showed a higher association between PM_2.5_ and violence (Figure 3), although the statistical evidence (Wald test for the effect modification) was weak. Especially, we could not find statistically meaningful differences between high and low GRDP areas. We have added the related texts in the Results section as below:

**(Lines 255-262 on Pages 13-14)**

Figure 3 shows the association between PM_2.5_ and violence in the total population and by sub-group. In the total population, the association between PM_2.5_ and violence was evident with an OR of 1.07 with 95% CI: 1.02–1.12. The association was weakly higher in rural areas (OR: 1.23, 95% CI: 1.02–1.48) than in urban areas (OR: 1.06, 95% CI: 0.99–1.12) (Wald test p-value: 0.096). Further, the association between PM_2.5_ and violence was statistically pronounced in cold seasons (OR: 1.08, 95% CI: 1.02–1.14) compared to warm seasons (1.05, 0.96–1.15), and the association was more pronounced in people aged 65 (OR:1.33, 95% CI: 1.04–1.71) or older than those aged 64 or less in cold seasons (Wald test p-value: 0.042).

However, because of the limited data availability (the surveillance system database used in this study does not include the information on enrollees’ occupation and income status), we could not consider an individual's income level and we have addressed this issue as a major limitation in the discussion section:

**(Lines 378-381 on Page 24)**

Third, due to the limited data availability, we were unable to explore the associations between PM_2.5_ and violence by individual socioeconomic factors, such as occupation or income levels, although these socioeconomic factors could be substantially associated with violence.

**#2.6. I am also curious if the authors can consider subtypes of violence. If possible, I recommend adding an analysis by types of violence. Furthermore, while the supplementary file includes some details, the authors should specify the ICD codes used for the classification of violence cases.**

**[Response]** We truly appreciate your insightful comment. We have added descriptive statistics for the detailed classification of mechanisms according to ICD-9 codes in Table S5. However, the majority of cases were due to collisions and the other categories accounted for less than 10%. Therefore, there were difficulties in performing stratified analyses by subtype of violence due to insufficient sample size for the statistical analysis, and we have addressed this issue in the Discussion section as a major limitation.

**(Lines 385-391 on Page 24)**

In addition, although we reported the descriptive statistics on subtypes of violence based on diagnostic code, we could not present the stratified association between PM_2.5_ and violence by subtype because of the insufficient sample size of each subtype: the majority of violence cases were due to collisions (around 90%). It would be helpful to improve the understanding of the underlying mechanisms between air pollution and violence if future studies could examine the types of violence with larger prospective cohort data.

**#2.7. Table 2 and Figure 3: The authors claim that the excess burden for those aged 0-64 is higher than that for those aged 65 and above, but this varies considerably by season. For instance, in the cold season, the odds ratio and excess burden for the 65+ age group are much higher. I would be interested to know the authors’ thoughts on this discrepancy.**

**[Response]** We are thankful for your feedback. First, the higher excess numbers related to PM_2.5_ in people aged 64 or less (Table 2) were due to the large number of violence cases in the population (Table 1); stratified results (Figure 3) show that the associations between PM_2.5_ and violence (odd ratio) were lower in people aged 64 or less than in those aged 65 or older across all seasons. Therefore, in accordance with your suggestion, we have tried to find plausible explanations for the difference in the association by age.

One possible explanation is that older people (65+ years), who may experience weakened immune systems, underlying conditions, and decline in physical function during the cold season, can be more vulnerable to exposure to air pollution and risk of injury during this season. Further, alcohol consumption, which is closely linked to violent behavior,^22^ tends to increase during the cold months. Cold weather and reduced sunlight, as well as holidays such as New Year’s and Christmas, are associated with higher alcohol intake.^23-25^ This is especially concerning for older adults (65+ years), as increased alcohol consumption can worsen pre-existing vulnerabilities to physical and mental health problems.^26^ While more research is needed to understand the differences in the effects of air pollution on violence by season and age group, we have added our hypotheses in the discussion section below:

**(Lines 352-361 on Page 23)**

Additionally, it is worth noting that in this study only those aged 65 years or older had a significantly higher risk during the cold season compared to all seasons, while the risk estimates for those aged 64 years or less did not change by season. Older adults, who may experience weakened immune systems, worsening of underlying conditions, and decline in physical function during the cold season, can be more vulnerable to exposure to air pollution and risk of injury during this season. Further, alcohol consumption, which is closely linked to violent behavior,^48^ tends to increase during the cold months; cold weather and reduced sunlight, as well as holidays such as New Year’s and Christmas, are associated with higher alcohol intake.^49-51^ This is especially concerning for older adults (65+ years), as increased alcohol consumption can worsen pre-existing vulnerabilities to physical and mental health problems.^52^

**#2.8 Figure 2: The exposure-response curve suggests that concentrations below approximately 10 may not be associated with increased risk, indicating a potential threshold. What do the authors think about this? Given that linearity is observed at higher concentrations, I believe an additional analysis, such as piecewise regression considering a threshold, would be reasonable.**

**[Response]** Thank you for your comment, and we agree that checking the linearity is informative and needed. Following your suggestion, we fitted a piecewise regression analysis using 10 µg/m³ of PM_2.5_ as a threshold point and performed a likelihood ratio test (LRT) to check whether the piecewise model is statistically better than a model based on the linear association between PM_2.5_ and violence. We found that the piecewise regression model provides different point estimates of odds ratio: 0.947 (95% CI: 0.718–1.250) for PM_2.5_ levels below 10 µg/m³ and 1.125 (95% CI: 0.864–1.464) for levels above 10 µg/m³. However, the difference was not statistically significant, and the LRT test showed that there was no statistical difference between the piecewise and linear models (p-value: 0.3812).

Further, together with the improved interpretability of the linear model, we thought that using the linear model is better to show an “average” association between PM_2.5_ and violence. Thus, we carefully determined that using a linear exposure and response relationship is more suitable for this study. We would be grateful if you could consider our determination.

**#2.9. The authors emphasize WHO guidelines and NAAQS air quality standards. I recommend providing a more detailed explanation of these guidelines and discussing South Korea’s air pollution levels in the background section.**

**[Response]** We have added further explanations regarding the WHO guidelines and the NAAQS in the United States, as well as additional information about South Korea's air pollutant standards (KAS) in the main text as below (KAS was the same as the NAAQS).

**(Lines 214-223 on Page 11)**

Further, we assessed the excess violence burden associated with non-compliance with the current guidelines regarding PM_2.5_: the WHO guideline, the National Ambient Air Quality Standards (NAAQS) in the United States, and the Korean Air Quality Standards (KAS) in South Korea. These guidelines have been established to provide quantitative recommendations for air quality management, and exceeding these levels is associated with important risks to public health. Therefore, we calculated excess violence cases only for the subset of days with PM_2.5_ levels above (i.e. non-compliance) the WHO air quality guidelines (daily average PM_2.5_ > 15 μg/m^3^), and the NAAQS and KAS (daily average PM_2.5_ > 35 μg/m^3^), respectively, to illustrate the burden that could be prevented or reduced through compliance with the guidelines.

**#2.10. Lines 214-221, 302-305: The data used in this study is based on a sample of patients discharged from general hospitals. I believe this limitation should be considered when generalizing the study’s findings.**

**#2.11. The outcome definition relies on diagnosis codes, which may lead to an underestimation of actual violence incidence. The authors should consider this potential limitation.**

**[Response]** We appreciate your comment. While the survey dataset used in this study aimed to get population representativeness through specific sampling methods, we agree that there may still be potential bias, and caution is needed when interpreting the findings. Further, as you mentioned, we agree that the diagnosis code-based definition can provide the underestimation problem. Therefore, we have added the following limitations to the Discussion section:

**(Lines 381-485 on Page 26)**

Fourth, because our dataset only covered patients who were diagnosed admitted to, and discharged from the hospital, there was a limitation in capturing information on patients who were not admitted or who died before reaching the hospital. Also, the outcome definition relies on diagnosis codes, which may lead to an underestimation of the actual cases of violence.

**#2.12. Lines 245-245: It is unclear whether PM2.5 exposure increases the likelihood of aggressive behavior or the risk of being harmed. "Violence incidence" seems to refer to those harmed by violence, but the authors should clarify this distinction. Additionally, as social and environmental factors may play a larger role in violent events, it would be helpful to address these aspects in the discussion.**

**[Response]** We are grateful for your comment. Although this study was unable to clearly distinguish between victims or perpetrators of violence due to the limited data availability, it can be inferred that exposure to PM_2.5_ may affect violence and related mental health problems, increasing the risk of being involved in violent cases. We have added the related discussion in the text (lines 325-328 on page 22). Further, we agree that the term “violence incidence” can lead to misinterpretation. Thus, we omitted the word “incidence” across the manuscript.

**(Lines 325-328 on Page 22)**

Although we could not clearly distinguish between victims or perpetrators of violence due to data limitations, it can be inferred that exposure to PM_2.5_ may affect violence and related mental health problems, increasing the risk of being involved in violent incidents.

In addition, we agree that social factors can play important roles in violent events. Especially, regarding the roles of social factors, we described our additional data collections and stratified analyses by urban/rural and GRDP per 100,000 in response to #2.5. We would be grateful if you could read our previous response. We also agree that environmental factors also had critical roles in violence. Therefore, in accordance with your suggestion, we have attempted to summarize previous relevant studies and plausible mechanisms of environmental variables in the Discussion section.

**(Line 293-305 on Pages 20-21)**

In particular, intentional injuries such as violence are viewed as preventable,^34^ highlighting the importance of identifying modifiable risk factors. These include demographic factors (i.e., age, sex, and ethnicity), socioeconomic factors (i.e., education and employment status), and environmental factors (i.e., neighborhood safety and exposure to pollution). Furthermore, recent studies have reported consistent evidence that environmental factors might be associated with violence. Previous studies suggested that sudden weather changes (rising temperature, rain, an increase in humidity) can increase violent crimes, such as homicides, assaults, and sex offenses,^35^ and some theories (thermal or climatic discomfort can increase impulsivity and aggression, and changes in routine activities due to changing weather, such as outdoor events and social engagements) supports these results on weather and violence.^35,36^ In this context, the findings of this study are also anticipated to contribute valuable evidence for developing environmental epidemiological strategies aimed at violence prevention.

**Minor comments:
#2.13. Abstract, Line 34: I believe there is a misstatement regarding the estimated excess fraction of violence cases attributable to PM2.5. The confidence intervals and estimates are unclear.**

**[Response]** Thank you for your feedback. We have corrected the text as follows to clarify our estimates and confidence intervals:

**(Line 34-37 on Page 2)**

The estimated excess fraction of violence cases related to PM_2.5_ was 14.53% (95% CI: 4.54%–22.92%), and 6.42% (95% CI: 1.97%–10.26%) of the excess violence was related to non-compliance with the WHO guidelines (daily PM_2.5_ > 15 µg/m^3^).

**#2.14. I recommend that the authors provide a table of contents and page numbers for the supplementary file.**

**[Response]** Thank you for your comment. We added a table of contents and page numbers in the revised supplementary materials.

**#2.15. The authors should link specific paragraphs in the supplementary file to the appropriate text in the manuscript (e.g., "2. Air Pollution Prediction Models" should reference "Supplementary Text 2").**

**[Response]** We have revised all the related texts to link the sentences in the main text and the supplementary materials

**(Lines 128-130 on Page 7)**

Detailed information on the model is reported in the Supplementary Materials, “1. Air pollution prediction models” and Supplementary Table 1 (Table S1).

**(Lines 133-135 on Pages 7-8)**

Supplementary Table S2-S3 displays the prediction performance of the PM_2.5_ ensemble prediction model used in this study.

**(Lines 237-241 on Page 12)**

Annually, approximately 544 to 612 cases of violence were reported in South Korea (Supplementary Table S4). Collisions accounted for the highest proportion of injury mechanisms at 88.7% (Supplementary Table S5). For treatment outcomes, complete and partial recovery was the most common, occurring in 96.16% of cases (Supplementary Table S6).

**#2.16. Some results are missing odds ratios and 95% confidence intervals. I recommend addressing this (e.g., Lines 190-195).**

**[Response]** We have added several missed values in the result section following your suggestion.

**#2.17. Lines 211-212: This sentence could be moved to the Statistical Analysis or Sensitivity Analysis section.**

**[Response]** We revised that sentence in accordance with your suggestion:

**(Lines 226-228 on Page 12)**

**Sensitivity analysis**

We performed a series of sensitivity analyses to examine whether our results were consistent with additional different modeling specifications in the total population.

**(Lines 279-280 on Page 20, Results part)**

Lastly, the results of sensitivity analyses showed that our main results were consistent with different modeling selections (Table S8).

**#2.18 Although Figure 1 provides PM2.5 levels, I recommend including the mean concentration of PM2.5 in the main text.**

**[Response]** We have added the national annual average concentration of PM_2.5_ in Table S1 and main text as follows:

**(Lines 241-244 on Page 12)**

Figure 1 shows geographical distributions of average PM_2.5_ during the study period. The national annual average concentration of PM_2.5_ was 23.71 µg/m³, with the lowest level recorded in 2018 at 21.71 µg/m³ and the highest in 2015 at 25.74 µg/m³ (Supplementary Table S1).

**Reviewer #3: 1. In Table 1, the age is categorized as 0-19, 20-39, 40-64, 65-79, 80+, but I wonder if the actual analysis uses 0-64, 65+. I recommend analyzing the age according to Table**

**#3.1. If you think that the analysis results by subdividing the age categories are not better than the existing analysis results, please express the age as under 65 and over 65 in Table 1.
[Response]** We are grateful for your comment and agree with your suggestion. We have added descriptive information on individuals aged under 65 and 65 or older, respectively. Please see the revised Table 1 below. However, we would like to request your understanding that Reviewer 2 suggested additional analyses with more subdivided age groups, thus we have performed additional subgroup analyses (Table S7) using more specific age groups reported in Table 1.

**Table 1. Descriptive information on violence cases among Korea National Hospital Discharge In-depth Injury Survey Data during the study period (2015–2019) in South Korea.** Holidays include Official public holidays in Korea. We classified the study districts (si/gun/gu) into urban (si and gu) and rural (gun) districts. GRDP High and Low areas are separated by a median value.

|  |  | **N** | **%** |
| --- | --- | --- | --- |
| **Total** |  | 2867 | 100 |
| **Sex** | **Males** | 1937 | 67.6 |
|  | **Females** | 930 | 32.4 |
| **Age** | **0-64 years** | 2660 | 92.8 |
|  | 0-19 years | 442 | 15.4 |
|  | 20-39 years | 1023 | 35.7 |
|  | 40-64 years | 1195 | 41.7 |
|  | **65+ years** | 207 | 7.2 |
|  | 65-79 years | 174 | 6.1 |
|  | 80+ years | 33 | 1.2 |
| **Holiday** | **Yes** | 106 | 3.7 |
|  | **No** | 2761 | 96.3 |
| **Urban/Rural** | **Urban** | 2568 | 89.6 |
|  | **Rural** | 299 | 10.4 |
| **GRDP per person** | **High** | 2095 | 73.1 |
|  | **Low** | 772 | 26.9 |

**#3.2. There are treatment results in the data. Please classify them as follows and calculate the risk of no hope and death.
- Unknown means missing.
- No hope and death are one category.
- Remaining results are one category.**

**[Response]** Thank you for your meaningful comment. We have added the descriptive information by treatment results in Table S6 in accordance with your suggestion. However, because the majority (above 96%) of cases were “Recovered (Complete recovery and Partial recovery)”, we were unable to perform stratified analysis by treatment results. We appreciate your understanding.

**Table S6. Descriptive information on violence cases among Korea National Hospital Discharge In-depth Injury Survey Data during the study period (2015–2019) in South Korea by season and treatment result.**

| **Treatment result** | **Total season** | **Warm seasons** | **Cold**  **seasons** |
| --- | --- | --- | --- |
| **Total** | 2867 (100%) | 1549 (100%) | 1318 (100%) |
| Recovered (Complete recovery and Partial recovery) | 2757 (96.16%) | 1492 (96.32%) | 1265 (95.98%) |
| Diagnosed but not treated | 48 (1.67%) | 24 (1.55%) | 24 (1.82%) |
| Not recovered (No improvement) | 45 (1.57%) | 26 (1.68%) | 19 (1.44%) |
| Discharged due to death | 16 (0.56%) | 7 (0.45%) | 9 (0.68%) |
| Others | 1 (0.03%) | 0 (0.00%) | 1 (0.08%) |

**References for Responses**

1.	Kim Y, Oh J, Kim S, et al. Relationship between short-term ozone exposure, cause-specific mortality, and high-risk populations: A nationwide, time-stratified, case-crossover study. *Environmental Research* 2024; **261**: 119712.

2.	Min J, Lee W, Kang D-H, et al. Air pollution and acute kidney injury with comorbid disease: A nationwide case-crossover study in South Korea. *Environmental Research* 2024; **260**: 119608.

3.	Di Q, Wang Y, Zanobetti A, et al. Air Pollution and Mortality in the Medicare Population. *New England Journal of Medicine* 2017; **376**(26): 2513-22.

4.	Kioumourtzoglou M-A, Schwartz J, James P, Dominici F, Zanobetti A. PM2. 5 and mortality in 207 US cities: modification by temperature and city characteristics. *Epidemiology* 2016; **27**(2): 221-7.

5.	Zhou L, Wang Y, Wang Q, et al. The interactive effects of extreme temperatures and PM2. 5 pollution on mortalities in Jiangsu Province, China. *Scientific Reports* 2023; **13**(1): 9479.

6.	Lee W, Prifti K, Kim H, et al. Short-term exposure to air pollution and attributable risk of kidney diseases: a nationwide time-series study. *Epidemiology* 2022; **33**(1): 17-24.

7.	Janssen N, Fischer P, Marra M, Ameling C, Cassee F. Short-term effects of PM2. 5, PM10 and PM2. 5–10 on daily mortality in the Netherlands. *Science of the total environment* 2013; **463**: 20-6.

8.	Gasparrini A, Guo Y, Hashizume M, et al. Mortality risk attributable to high and low ambient temperature: a multicountry observational study. *The lancet* 2015; **386**(9991): 369-75.

9.	Lee W, Kim Y, Sera F, et al. Projections of excess mortality related to diurnal temperature range under climate change scenarios: a multi-country modelling study. *The Lancet Planetary Health* 2020; **4**(11): e512-e21.

10.	Gasparrini A, Leone M. Attributable risk from distributed lag models. *BMC Medical Research Methodology* 2014; **14**(1): 55.

11.	Wei Y, Wang Y, Di Q, et al. Short term exposure to fine particulate matter and hospital admission risks and costs in the Medicare population: time stratified, case crossover study. *BMJ* 2019; **367**: l6258.

12.	Lee W, Wu X, Heo S, et al. Air Pollution and Acute Kidney Injury in the U.S. Medicare Population: A Longitudinal Cohort Study. *Environmental Health Perspectives* 2023; **131**(4): 047008.

13.	Sun Y, Milando CW, Spangler KR, et al. Short term exposure to low level ambient fine particulate matter and natural cause, cardiovascular, and respiratory morbidity among US adults with health insurance: case time series study. *BMJ* 2024; **384**: e076322.

14.	Wellenius GA, Burger MR, Coull BA, et al. Ambient Air Pollution and the Risk of Acute Ischemic Stroke. *Archives of Internal Medicine* 2012; **172**(3): 229-34.

15.	Zanobetti A, Dominici F, Wang Y, Schwartz JD. A national case-crossover analysis of the short-term effect of PM2.5 on hospitalizations and mortality in subjects with diabetes and neurological disorders. *Environmental Health* 2014; **13**(1): 38.

16.	Sun S, Weinberger KR, Nori-Sarma A, et al. Ambient heat and risks of emergency department visits among adults in the United States: time stratified case crossover study. *BMJ* 2021; **375**: e065653.

17.	Park J, Kim A, Kim Y, et al. Association between heat and hospital admissions in people with disabilities in South Korea: a nationwide, case-crossover study. *The Lancet Planetary Health* 2024; **8**(4): e217-e24.

18.	Lee W, Choi M, Bell ML, et al. Effects of urbanization on vulnerability to heat-related mortality in urban and rural areas in South Korea: a nationwide district-level time-series study. *International Journal of Epidemiology* 2021; **51**(1): 111-21.

19.	Jang H, Lee W, Kim Y-o, Kim H. Suicide rate and social environment characteristics in South Korea: the roles of socioeconomic, demographic, urbanicity, general health behaviors, and other environmental factors on suicide rate. *BMC Public Health* 2022; **22**(1): 410.

20.	Park J, Kim A, Bell ML, Kim H, Lee W. Heat and hospital admission via the emergency department for people with intellectual disability, autism, and mental disorders in South Korea: a nationwide, time-stratified, case-crossover study. *The Lancet Psychiatry* 2024; **11**(5): 359-67.

21.	Kang C, Park C, Lee W, et al. Heatwave-Related Mortality Risk and the Risk-Based Definition of Heat Wave in South Korea: A Nationwide Time-Series Study for 2011–2017. *International Journal of Environmental Research and Public Health* 2020; **17**(16): 5720.

22.	Parker RN, Auerhahn K. Alcohol, drugs, and violence. *Annual review of sociology* 1998; **24**(1): 291-311.

23.	Ventura‐Cots M, Watts AE, Cruz‐Lemini M, et al. Colder weather and fewer sunlight hours increase alcohol consumption and alcoholic cirrhosis worldwide. *Hepatology* 2019; **69**(5): 1916-30.

24.	Heacock RM, Capodilupo ER, Czeisler MÉ, et al. Sleep and Alcohol Use Patterns During Federal Holidays in the United States. *medRxiv* 2021: 2021.11. 04.21264353.

25.	Lloyd B, Matthews S, Livingston M, Jayasekara H, Smith K. Alcohol intoxication in the context of major public holidays, sporting and social events: a time–series analysis in M elbourne, A ustralia, 2000–2009. *Addiction* 2013; **108**(4): 701-9.

26.	Sacco P, Bucholz KK, Spitznagel EL. Alcohol use among older adults in the National Epidemiologic Survey on Alcohol and Related Conditions: A latent class analysis. *Journal of studies on alcohol and drugs* 2009; **70**(6): 829-38.
