# Supplementary material for: Association between fine particulate matter (PM2.5) and violence cases in South Korea: A nationwide time-stratified care-crossover study
Source: PLoS One. 2024 Dec 17;19(12):e0315914. doi: 10.1371/journal.pone.0315914 (PMC11651589; doi:10.1371/journal.pone.0315914)
Supplement: Attachment — Submitted filename: Responses to Reviewers_20241127.docx [file pone.0315914.s003.docx]

**Dear Editor,**

We sincerely thank the reviewers for their insightful comments on our manuscript. We have carefully revised the manuscript and responded to each comment point by point below.

**Journal Requirements:**

**Please review your reference list to ensure that it is complete and correct. If you have cited papers that have been retracted, please include the rationale for doing so in the manuscript text, or remove these references and replace them with relevant current references. Any changes to the reference list should be mentioned in the rebuttal letter that accompanies your revised manuscript. If you need to cite a retracted article, indicate the article’s retracted status in the References list and also include a citation and full reference for the retraction notice.**

**[Response]** We truly appreciate your careful check and would like to express our apologies. We could not find that there was an error in the Endnote we used, and thus previous reference list was incorrect. Once again, we apologize for the confusion. In accordance with the criteria you mentioned, we have revised and double-checked references, and we found that the current references in the revised manuscript are correct. Please the modifications we performed below:

**(List of the reference modifications)**

**#1.** “*14.	Burkhardt J, Bayham J, Wilson A, et al. The relationship between monthly air pollution and violent crime across the United States. Journal of Environmental Economics and Policy 2020; 9(2): 188-205*”. This study includes all countries of the United States. Thus, this is not a suitable reference to our description of “many existing studies were conducted in the restricted areas”. We replaced this reference as the “***Nguyen A-M, Malig BJ, Basu R. The association between ozone and fine particles and mental health-related emergency department visits in California, 2005–2013. PloS one 2021; 16(4): e0249675****.*”, which is the study conducted in California with monitored air pollutants.

**#2**. We deleted previous References 27-30 because they have similar information (why the time-stratified case-crossover design is suitable to evaluate the impacts of short-term exposure to environmental stressors and health outcomes), compared to previous References 21-26.

**#3.** (Lines 174). We found the error in the reference number: the previous manuscript had a “13-16” reference, which is incorrect. We have revised it with corrected references.

**#4.** (Lines 217) We found the error in the reference number. The previous manuscript had a “12, 17-20” reference, which is incorrect. We have revised it with a corrected reference showing the importance of the health impacts of low PM_2.5_ exposures.

**#5.** (Lines 232) We also found that there was an error in the reference number regarding the Monte Carlo simulation. We have revised it with the related references.

**#6.** (Line 110) We have added the references regarding the Neyman allocation method.

**Reponses to Reviewers**

**Reviewer #1: The authors have addressed most of my concerns.**

**#1.1. Although there is a possibility of misinterpretation for “excessive burden due to PM2.5” but I understand that the method is frequently used.**

**[Response]** We truly appreciate your understanding. As you mentioned, we recognized that the expression “excess burden due to” has been widely used in the relevant studies ^1-4^, even though this word has a possibility of misinterpretation related to causality or conceptual confusion between excess risk (RR_1_-RR_2_) and attributable risk ([RR_1_-RR_2_]/RR_1_). We tried to use the attributable risk as a measure of the proportion of the outcome occurrence that can be attributed to ambient PM_2.5_ exposures, thus this definition can be expressed by estimating excess risk as [RR_1_-RR_2_]/RR_1_. This formula provides the proportion of the excess risk of outcome that can be attributed to the exposure.^5^

Thus, we cautiously thought that this concept was aligned with the measurement we would like to estimate. Nonetheless, to avoid potential confusion and clarify the concept we adopted, we have revised all related terms to “excess burden/number attributable to PM_2.5_” across the manuscript. Once again, we are grateful for your generous understanding.

**Reviewer #2: I would like to thank the authors for their thoughtful responses to my concerns.**

**I have a few additional minor comments, and I would appreciate it if these could be considered.**

**#2.1. Line 95: "We obtained national data on hospital visits due to violence from 2015 to 2019 across247" I suggest correcting "across247" to "across 247".**

**[Response]** Thank you so much for your careful check. We have revised it.

**#2.2. Line 115: "Each recorded case also includes information on the patient’s sex, age, residential address (ZIP code), date of hospitalization". While the residential address in South Korea can be considered similar to a ZIP code, it is not identical. I recommend modifying this expression to reflect that they are not exactly the same.**

**[Response]** Thank you for pointing out the important point. It was our mistake. It was not a Zip code. It should be the district called “si-gun-gu” in Korean. We sincerely apologize for our mistake. Please see our modification below:

**(Lines 115-116 on Page 7)**

Each recorded case also includes information on the patient’s sex, age, residential address (districts, “*si/gun/gu*” in Korean),

**#2.3. Regarding Excess violence, typically, excess mortality or excess hospitalization is calculated using relative risk (RR). In cases where the disease incidence is low (rare disease), OR approximates RR, so using OR as a substitute for RR is not a significant concern. However, the authors should consider adding a brief explanation in the text to clarify this relationship between OR and RR.**

**[Response]** We genuinely appreciate your suggestion. Yes, as you mentioned, the odd ratio (OR) can be regarded as an approximated estimate of the relative risk (RR) when the outcome risk is low in both groups (in general, 20% or less).^6^ The total number of outcomes (violence cases) was 2,867 cases during the find years (2015 to 2019), and when the total number of investigation cases of the Korea National Hospital Discharge In-depth Injury Survey (sampled 150,000 to 300,000; see lines 102), the probability of our outcome was substantially small. Thus, as you said, we also thought that our OR estimates could be good approximated values of RRs that are needed to calculate the attributable fraction. In accordance with your suggestion, we have added the related text in the Method section.

**(Lines 204-212 on Page 11)**

Herein, the OR estimates were used as the approximated values of relative risk (RR) that were originally used to calculate the attributable risk estimates. When the proportion of the outcome onset is substantially small (in general, 20% or less), the OR estimates can be regarded as an approximated estimate of the RR estimates ^35^. In this study, the total number of outcomes (violence cases) was 2,867 cases during the find years (2015 to 2019), and when the total number of investigation cases of the Korea National Hospital Discharge In-depth Injury Survey (sampled 150,000 to 300,000; see lines 101). Therefore, the probability of our outcome was substantially small. Thus, we used OR estimates as the approximated values of RR estimates in this study.

**References for the responses**

1.	Gasparrini A, Guo Y, Hashizume M, et al. Mortality risk attributable to high and low ambient temperature: a multicountry observational study. *The Lancet* 2015; **386**(9991): 369-75.

2.	Lee W, Prifti K, Kim H, et al. Short-term Exposure to Air Pollution and Attributable Risk of Kidney Diseases: A Nationwide Time-series Study. *Epidemiology* 2022; **33**(1).

3.	Min J, Kang D-H, Kang C, et al. Fluctuating risk of acute kidney injury-related mortality for four weeks after exposure to air pollution: A multi-country time-series study in 6 countries. *Environment International* 2024; **183**: 108367.

4.	Weinberger KR, Harris D, Spangler KR, Zanobetti A, Wellenius GA. Estimating the number of excess deaths attributable to heat in 297 United States counties. *Environmental Epidemiology* 2020; **4**(3).

5.	Thelle DS, Laake P. Chapter 9 - Epidemiology. In: Laake P, Benestad HB, Olsen BR, eds. Research in Medical and Biological Sciences (Second Edition). Amsterdam: Academic Press; 2015: 275-320.

6.	Cook A, Sheikh A. Descriptive statistics (Part 2): Interpreting study results. *Primary Care Respiratory Journal* 2000; **8**(1): 16-7.
